# Associations between Childhood Abuse, Resilience, Mindfulness, and Waterpipe Smoking: Implications for Cessation Interventions

**DOI:** 10.1155/2021/6648779

**Published:** 2021-08-02

**Authors:** Mohammadreza Naghavi, Nouzar Nakhaee

**Affiliations:** ^1^Social Determinants of Health Research Center, Institute of Futures Studies in Health, Kerman University of Medical Sciences, Kerman, Iran; ^2^Research Center for Health Services Management, Institute of Futures Studies in Health, Kerman University of Medical Sciences, Kerman, Iran

## Abstract

**Introduction:**

Recent research has established a link between childhood abuse and later drug abuse. For waterpipe smoking (WPS), such a role has not been adequately clarified.

**Aims:**

To explore the mediating effect of resilience and mindfulness on the association between childhood abuse and current WPS among college students.

**Methods:**

A cross-sectional study was conducted among a consecutive sample (*n* = 776) of college students in Kerman, Iran. The Adverse Childhood Experiences Abuse Short Form, the 14-item Resilience Scale, and the Freiburg Mindfulness Inventory were used. Structural equation modeling was used to examine the complex associations between variables.

**Results:**

Nearly 95% of participants were aged between 18 and 27 years, and the mean (SD) age of students was 22.2 (3.1). Most of them were single (84.4), and 52.7% were female. Prevalence of lifetime and current WPS among students was 49.6% and 33.4%, respectively. Less than one-third (*n* = 228) of lifetime users first tried smoking by the age of 18. The risk of current WPS was significantly higher in males than females (*β* = 0.25, *P* < 0.001). Childhood abuse was directly associated with current WPS (*β* = 0.20, *P* < 0.001) and resilience (*β* = −0.12, *P* < 0.05). Adverse childhood experiences were also indirectly (mediated by the effect of the resilience, path coefficient = 0.06, *P* < 0.001) associated with the risk of WPS. No relationship was seen between trait mindfulness and current WPS (*β* = −0.02, *P* = 0.393). Resilience was negatively associated with current WPS (*β* = −0.47, *P* < 0.001).

**Conclusion:**

The study revealed the potential importance of childhood abuse and low resilience as risk factors precipitating the onset of WPS. Further studies are warranted to examine the implications of this study for quitting WPS.

## 1. Background

Waterpipe smoking (WPS) as a worldwide growing health hazard has several impacts on the health of communities [1]. It has detrimental effects on the cardiovascular system and lungs and is linked to many adverse health effects as cigarette smoking such as cancers [1]. In recent years, a worldwide surge in WPS popularity especially among adolescents is evident [2]. A major reason behind this popularity is misconceptions about WPS ([3]), conceptions like passing the smoke through water reduces harmful effects or considering WPS as a nonaddictive form of tobacco use [3].

As a whole, the prevalence of WPS is higher in the Eastern Mediterranean region compared to the other regions of the world, though in some of the European countries it has become close to the Middle East countries [2]. In a systematic review, the highest figures of waterpipe ever use were seen among Lebanese college students (i.e., 65.3%), and the corresponding figure regarding regular use of waterpipe among college students was reported from Iran (i.e., 16.2%) [4]. When students were asked about reasons for initiation of WPS, they gave reasons such as for fun, curiosity, peer influence, overcoming anxiety and stress, and dealing with depression and anger [3, 5, 6]. An Arab study concluded that mentally ill patients smoke waterpipe 1.5 times more than the general population [7].

A cross-sectional study showed that WPS may be moderately associated with common mental health problems such as anxiety, depression, and tremendous stress among college students [8]. Despite the health hazards of WPS and its addiction potential, there are few studies on WPS cessation intervention [5].

It seems that each factor that has a potential protective role against mental ill-being may be utilized in designing cigarette smoking (and perhaps WPS) prevention and cessation intervention programs ([9]). One of the contributing factors, which its relationship with mental health status has been well documented, is trait resilience [10]. According to the American Psychological Association, resilience refers to the process of being able to adapt well in the face of significant sources of stress or trauma [11]. Resilience has not only been shown to be a protective factor against cigarette smoking but also building it is now accepted as a means to smoking cessation [12]. To the best of our knowledge, such an effect has not been studied regarding WPS. As with resilience, dispositional mindfulness has a positive relationship with psychological health ([13]). Mindfulness refers to the innate propensity to be aware of our thoughts and feelings in the present moment in a nonjudgmental manner [13]. Recent findings suggest that dispositional mindfulness tends to be lower among adolescents and adults with a history of childhood abuse [14]. There is also a negative relationship between trait mindfulness and substance use behaviors including cigarette smoking [15], although such a relationship has not been addressed regarding WPS in the literature.

There is a growing body of literature indicating that adverse childhood experiences (ACEs) could play a deleterious role and increase the risk of mental health problems and drug abuse and cigarette smoking in later life [16, 17]. ACE refers to potentially traumatic events occurring before age 18.

Owing to the long-term impact of childhood adversity on different aspects of mental health including tobacco smoking, clarifying the pathways that childhood abuse may impose its consequences on the risk of WPS would be informative. Resilience and mindfulness are the two deeply intertwined resources that are related to different aspects of mental health such as substance use [10, 13]. Considering the powerful combination of resilience and mindfulness in promoting the psychological wellbeing of individuals, it would be very important that we could depict the pathways and relationships that may exist between one possible major risk factor for WPS (i.e., childhood abuse) and the two possible and correlated mitigating factors (i.e., resilience and mindfulness). Estimating and evaluating a model including all of these three domains would have implications for planning effective smoking cessation interventions [18].

The mediating roles of resilience ([19]) and mindfulness [20] on the causal relationship between ACEs and drug and alcohol abuse have been shown in recent studies, but to the best of our knowledge, such a relationship has not been examined regarding WPS. In this study, we aimed to identify the mediating roles of resilience and mindfulness between ACE and current WPS among a sample of college students hoping it may illuminate areas for cessation programs.

## 2. Materials and Methods

### 2.1. Setting and Participants

This cross-sectional study was conducted in 2019 at the Kerman University of Medical Sciences. It is located in Kerman city as the center of the largest province of Iran. Students were enrolled in the study using convenience sampling. Questionnaires were completed anonymously in the classrooms. A researcher distributed the self-administered questionnaires among students. Before leaving the classroom, a sealed box was provided to students to drop the completed questionnaires.

### 2.2. Measures

We used five well-validated questionnaires in this study: (1) sociodemographic questionnaire, (2) WPS status questionnaire [6], (3) Adverse Childhood Experiences Abuse Short Form (ACE-ASF) [21], (4) 14-item Resilience Scale (RS-14) for measuring resilience [22], and (5) Freiburg Mindfulness Inventory (FMI) [23].

### 2.3. Sociodemographic Questionnaire

Sociodemographic items included age, gender, marital status, degree level, living place, and study year.

### 2.4. WPS Status Questionnaire

It consisted of items regarding lifetime (ever) and current (past 30 days) use of waterpipe and age of WPS initiation Accordingly, the subjects could fall in either of the three categories: current smokers, ever smoker, and nonsmoker. This part of the questionnaire was well validated in the Iranian setting [6]. The current use of cigarettes was also asked.

### 2.5. The Adverse Childhood Experiences Abuse Short Form (ACE-ASF)

It is a short version of the Adverse Childhood Experiences International Questionnaire (ACE-IQ) consisting of eight items which solely measure child abuse in three domains of physical abuse (two items), emotional abuse (two items), and sexual abuse (four items) [21]. The response category of each item ranged from “I refuse to answer” to “many times.” Examples of items in the ACE-ASF include “Did a parent, guardian, or other household member yell, scream or swear at you, insult, or humiliate you?” and “Did a parent, guardian, or other household member spank, slap, kick, punch, or beat you up?” The ACE score was calculated using the frequency version [21]. The psychometric properties of the Persian version of the questionnaire have been confirmed [24]. Cronbach's alpha of the questionnaire was 0.81. The test-retest reliability coefficient of the Persian version of the tool was 0.73, and the factor loadings of all items were above 0.69 [24].

### 2.6. The 14-Item Resilience Scale (RS–14)

RS-14 is a self-rating questionnaire measuring resilience [23]. It consists of 14 questions measured on a 7-point Likert scale (1: strongly disagree to 7: strongly agree). Sample items on RS-14 include “I am friends with myself” and “I keep interested in things.”. The Persian version of RS-14 has been shown to have good reliability and validity [22]. Cronbach's alpha for the total scale was 0.87. The test-retest intraclass correlation coefficient (ICC) was 0.90 [22]. Concurrent and predictive validity of RS-14 was confirmed by high correlations with other resilience questionnaires and variables such as anxiety, depression, emotions, and coping along time [23, 25].

### 2.7. The Freiburg Mindfulness Inventory (FMI)

It is a brief questionnaire consisting of 14 items using the Likert scale (from “rarely” coded 1 to “almost always” coded 4). It measures mindfulness in generalized contexts where background knowledge regarding mindfulness may not be expected [26]. Sample items include “I accept unpleasant experience” and “I see my mistakes and difficulties without judging them.” In the validation study, it was shown that higher scores of FMI were predictive of lower psychological distress, and also, the questionnaire developers concluded that FMI could be used in those without previous meditation experience [26]. The Iranian version of the inventory has been shown to have acceptable psychometric properties ([27]). The Persian version had Cronbach's alpha of 0.77, and the ICC of test-retest reliability was 0.83.

### 2.8. Ethical Considerations

Before distributing the questionnaires, the aim of the study was explained to the students and they were assured about the anonymity and untraceability of their responses. Based on EC permission, verbal consent was obtained from the students and their parents, and they were reassured that their participation would be voluntary.

The ethics committee of the Kerman University of Medical Sciences approved the protocol of the study.

### 2.9. Analysis

We used descriptive statistics including means, frequencies, and percentages. The internal consistency of the questionnaire was calculated using Cronbach's alpha. A structural equation modeling (SEM) was applied to test whether our conceptual model was able to explain the observed relationships between variables and to analyze the mediators involved in reducing the impact of ACEs on current WPS. At first, the model was designed with a well-defined question. Current WPS was entered as a manifest variable, as it was measured by a single dichotomous (yes/no) item. ACEs, resilience, and mindfulness were entered as latent variables. Then, confirmatory factor analysis was used to test the measurement model. Model fitness was evaluated using four indices including *χ*^2^/df (ratio of *χ*^2^ to degrees of freedom), Comparative Fit Index (CFI), Root Mean Square Error Approximation (RMSEA), and Tucker-Lewis index (TLI). *P* values less than 0.05 were considered statistically significant. SPSS 20 and AMOS 24 were used to analyze the data.

## 3. Results

The overall response rate was 94.6% (776 of 820). Of the respondents, 94.6% were aged between 18 and 27 years, and the mean (SD) of respondents was 22.2 (3.1). Most of the students were single (84.4%). Other baseline variables are shown in Table 1.

Prevalence of ever and current WPS among college students was 49.6% (*n* = 385) and 33.4% (*n* = 259), respectively. Nearly 60% (*n* = 228) of lifetime users initiated WPS before age 18 years. Current cigarette smokers consisted 23.7% (*n* = 184) of the sample.

The mean (SD) of resilience and mindfulness score of subjects was 65.3 (19.7) and 33.3 (6.0), respectively. Nearly one-third (30.3%) of participants reported at least one type of child abuse, 25.0% reported two types, and 22.3% reported all of the three types of abuse.

**Figure 1:**
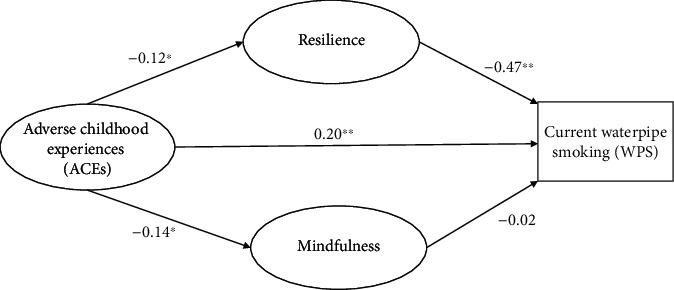
The mediating role of resilience and mindfulness on the relationship between previous adverse childhood experiences and current waterpipe smoking (^∗^*P* < 0.05, ^∗∗^*P* < 0.001). The estimate of the indirect effect of resilience on WPS was −0.12∗−0.47 = 0.06.

The pathway from childhood abuse, resilience, mindfulness, age, and gender to outcome (i.e., current WPS) was further analyzed by SEM modeling.  Figure 1 depicts the structural equation model with standardized path coefficients (with a maximum of 1 and a minimum of −1). This model had excellent fit (*χ*^2^/df = 2.95, CFI = 0.98, RMSEA = 0.03, and TLI = 0.98). The path coefficient of mindfulness to WPS (-0.02) was not significant (*P* = 0.393). The indirect effect of ACEs and WPS through mindfulness was not significant (path coefficient = 0.003). ACEs were directly (path coefficient = 0.20, *P* < 0.001) and indirectly (mediated by the effect of the resilience, path coefficient = 0.06, *P* < 0.001) associated with risk of WPS. Male gender was associated with a higher risk of WPS (standardized coefficient = 0.25, *P* < 0.001). Males were at higher risk of childhood trauma (standardized coefficient = 0.23, *P* < 0.001). Resilience had a significant protective effect against WPS (standardized coefficient = −0.47, *P* < 0.001).  Table 2 shows the path coefficient of all of the variables included in the model.

## 4. Discussion

Child abuse imposes several adverse impacts on physical and psychosocial aspects of adolescents' health, and it has been well known as a risk factor for drug abuse and cigarette smoking among adolescents [16, 17]. The relationship between childhood trauma and resilience and mindfulness as protective factors of alcohol abuse has been revealed in recent studies [19, 20]. Despite these research achievements, little work has been devoted to the relationship of WPS with ACEs and mediating role of resilience and mindfulness in this relationship. This study is the first report showing the interrelationship between these factors and WPS to be utilized in designing WPS cessation activities.

There is ample evidence that child abuse before age 18 years is associated with higher levels of drug use and cigarette smoking among adolescents including college students [16, 28, 29]. Our results showed that such a relationship is evident in waterpipe smokers too. It has been hypothesized that the link between ACEs and tobacco use is mediated by trait anxiety and mental illness [30]. Childhood abuse also indirectly increased the probability of WPS through decreased resilience, and that resilience directly increased the likelihood of WPS. So, an alternative pathway from childhood abuse to impaired resilience and subsequently to WPS results in even greater smoking risk. The role of resilience in preventing and quitting cigarette smoking has recently been more evident in the literature [18], but such evidence is lacking regarding WPS. Resilience gives adolescents the ability to adapt to and recover from difficult situations and in this way enables them not to turn to unhealthy coping mechanisms, such as smoking [31]. Loneliness has been known as a promoter of smoking in the literature [32] because students can gain a sense of belonging through WPS (Lee et al. [33]. Students who are far from their homes have a lonely feeling, and this may decrease their resilience. So, resilience in this group could play a major role in the prevention of WPS. Since childhood adversity showed a negative impact on resilience, WPS may induce a sense of belonging in lonely students and in this way make them more prone to WPS. In the field of cigarette smoking cessation, strengthening both internal (e.g., problem-solving skills and coping skills) and external (e.g., families and communities) factors of resilience should be considered. In this research, we found that resilience may be regarded as a component of WPS prevention programs too.

SEM depicted that those experiencing child abuse exhibited lower levels of trait mindfulness which is consistent with that of Brett et al. demonstrating an inverse relationship between mindfulness and history of childhood adversity (Brett et al. [20]. However, contrary to our initial expectation, our model showed that higher levels of mindfulness might not lead to a decrease in WPS. Leigh et al. even found a positive relationship between the FMI score of college students and smoking (i.e., students with higher levels of trait mindfulness were more likely to smoke cigarettes) [34]. There may be some explanations for our findings. First, most studies addressing the impact of mindfulness on drug use and/or smoking focus more on mindfulness training and practice than dispositional mindfulness, which is about inherent and baseline mindfulness [35]. Second, since measuring mindfulness with self-report items has raised some concerns, “many results of studies published so far have to be challenged” [36].

The structural results indicated that the risk of current WPS is higher in male students than female students in the Iranian context, which is in line with similar studies conducted in Islamic countries [6, 37]. The gender difference in the prevalence of WPS in Muslim countries may be due to social stigma and shame experienced by women and accordingly underreporting [38].

## 5. Limitations

This study has three limitations. We did not use probabilistic sampling so the generalizability of the findings should be interpreted with caution. Meanwhile, the taboo of childhood abuse, particularly sexual abuse in the Muslim context, may lead to underreporting. Since in naïve persons, the FMI score may not adequately measure the concept of mindfulness; it might be more preferable to use a more appropriate measurement tool.

## 6. Conclusion

Childhood abuse affects resilience, mindfulness, and WPS directly and also increases the risk of WPS by reducing resilience. So, childhood adversity could be included among other risk factors for WPS. Dispositional mindfulness was not related to WPS, which warrants further research. The direct mediating role of resilience may provide new and innovative ways for WPS cessation programs among college students irrespective of their childhood experiences.

## Figures and Tables

**Table 1:** Characteristics of college students (*n* = 776).

Variable		Frequency	%
Gender	Female	409	52.7
	Male	367	47.3
Marital status	Single	655	84.4
	Married	115	14.8
	Other	6	0.8
Degree level	Undergraduate	433	55.8
	Graduate	343	44.2
Living place	Dormitory	556	71.6
	With his/her family	189	24.4
	Renting house outside	31	4.0
Study year	1st year	315	40.6
	2nd year	169	21.8
	3rd year	126	16.2
	4th year or higher	166	21.4
Waterpipe smoking	Ever smoking	385	49.6
	Current smoking	259	33.4
Adverse childhood experiences	One type	235	30.3
	Two types	194	25.0
	All of the three types	173	22.3
Resilience score	Mean (SD)	65.3 (19.7)	
Mindfulness score	Mean (SD)	33.3 (6.0)	

**Table 2:** Estimated coefficients for the structural equation model.

Path	Standardized coefficient	SE	*P* value
ACEs⟶resilience	-0.12	0.090	0.011
ACEs⟶mindfulness	-0.14	0.027	<0.001
ACEs⟶WPS	0.20	0.025	<0.001
Resilience⟶WPS	-0.47	0.053	<0.001
Mindfulness⟶WPS	-0.02	0.032	0.393
Age⟶resilience	0.02	0.016	0.281
Age⟶mindfulness	0.02	0.005	0.549
Age⟶ACEs	-0.09	0.006	0.011
Age⟶WPS	-0.03	0.004	0.335
Gender⟶resilience	-0.03	0.103	0.440
Gender⟶mindfulness	-0.11	0.031	0.002
Gender⟶ACEs	0.23	0.040	<0.001
Gender⟶WPS	0.25	0.028	<0.001

## Data Availability

The data used to support the findings of this study are available from the corresponding author upon request.
